# Collagen fiber orientation disorder from H&E images is prognostic for early stage breast cancer: clinical trial validation

**DOI:** 10.1038/s41523-021-00310-z

**Published:** 2021-08-06

**Authors:** Haojia Li, Kaustav Bera, Paula Toro, PingFu Fu, Zelin Zhang, Cheng Lu, Michael Feldman, Shridar Ganesan, Lori J. Goldstein, Nancy E. Davidson, Akisha Glasgow, Aparna Harbhajanka, Hannah Gilmore, Anant Madabhushi

**Affiliations:** 1 Case Western Reserve University, Department of Biomedical Engineering, Cleveland, OH USA; 2 Case Western Reserve University, Department of Population and Quantitative Health Sciences, School of Medicine, Cleveland, OH USA; 3 Nanjing University of Information Science and Technology, Jiangsu Key Laboratory of Big Data Analysis Technique, Nanjing, China; 4 University of Pennsylvania Perelman School of Medicine, Philadelphia, PA USA; 5 Rutgers Cancer Institute of New Jersey, New Brunswick, NJ USA; 6 Fox Chase Cancer Center, Philadelphia, PA USA; 7 Fred Hutchinson Cancer Research Center, University of Washington, and Seattle Cancer Care Alliance, Seattle, WA USA; 8 University Hospitals Cleveland Medical Center, Cleveland, OH USA; 9 Louis Stokes Cleveland Veterans Administration Medical Center, Cleveland, OH USA

**Keywords:** Prognostic markers, Disease-free survival, Breast cancer, Cancer microenvironment

## Abstract

Collagen fiber organization has been found to be implicated in breast cancer prognosis. In this study, we evaluated whether computerized features of Collagen Fiber Orientation Disorder in Tumor-associated Stroma (CFOD-TS) on Hematoxylin & Eosin (H&E) slide images were prognostic of Disease Free Survival (DFS) in early stage Estrogen Receptor Positive (ER+) Invasive Breast Cancers (IBC). A Cox regression model named M_CFOD-TS_, was constructed using cohort S_t_ (*N* = 78) to predict DFS based on CFOD-TS features. The prognostic performance of M_CFOD-TS_ was validated on cohort S_v_ (*N* = 219), a prospective clinical trial dataset (ECOG 2197). M_CFOD-TS_ was prognostic of DFS in both S_t_ and S_v_, independent of clinicopathological variables. Additionally, the molecular pathways regarding cell cycle regulation were identified as being significantly associated with M_CFOD-TS_ derived risk scores. Our results also found that collagen fiber organization was more ordered in patients with short DFS. Our study provided a H&E image-based pipeline to derive a potential prognostic biomarker for early stage ER+ IBC without the need of special collagen staining or advanced microscopy techniques.

## Introduction

Breast cancer is the second leading cause of cancer death among women in the United States, with approximately 80% of these cancers being Estrogen Receptor Positive (ER+) and 64% being early stage (Stage I & II in Tumor, Node, Metastasis staging system)^1^. The standard treatment regimen for early stage ER+ Invasive Breast Cancer (IBC) patients is breast conserving surgery followed by hormonal therapy. While chemotherapy is able to significantly reduce the breast cancer associated mortality rate^2^, most early stage ER+ IBC patients do not receive added benefit from adjuvant chemotherapy^3^. Oncotype dx (Odx)^4^ is a 21-gene expression assay to assess the likelihood of cancer recurrence and the need for adjuvant chemotherapy for early stage IBC^5^. High Odx risk category indicates a high risk of recurrence and low Odx category suggests a low recurrence risk. The intermediate Odx category, however, has a more ambiguous prognostic/predictive meaning. While the Odx test is now routinely used in the clinical setting, Odx and other similar multi-gene assays tend to be time-consuming, high-cost, tissue-destructive and are not widely available in many countries.

Numerous studies have demonstrated that the interaction between tumor cells and extracellular matrix (ECM) plays a critical role in breast cancer progression and metastasis^6–9^. Collagen is the most abundant ECM component in breast cancer, which provides tensile strength and structural support to the tumor tissues^10^. Collagen fiber organization has been shown to be different between IBC and benign breast tumor, as well as between more and less aggressive IBC in both in-vivo/vitro models^7,11–14^. Using Second Harmonic Generation (SHG) based microscopy, Golaraei et al.^15^ observed an ultrastructure alteration in collagen fiber architecture in breast cancers, as compared to normal breast tissue. Friedl et al.^16^ observed that the focal sites of breast cancer cell micro-invasion often co-existed with stiff aligned collagen fibers. In addition, some studies^7,11,17^ have shown that organized collagen architecture in breast cancer was associated with a worse prognosis, as evidenced by SHG and Laser Scanning Microscopy (LSM) techniques.

Taken together, these studies strongly suggest that collagen fiber organization is associated with prognosis of breast cancer. However, the reported studies have employed advanced imaging techniques such as SHG or LSM microscopy^11,17–19^, techniques not routinely used in pathology labs or in clinical practice.

With the advent of digital pathology^20^, there has been substantial interest in exploring the role of computationally extracted quantitative histomorphometric attributes based on Hematoxylin & Eosin (H&E) stained Whole Slide Image (WSI) in diagnosis and prognosis across multiple cancer types^21–25^. In the context of breast cancer, histomorphometric features relating to measurements of shape, texture, and orientation of individual cancer nuclei both from the tumor epithelium^21–23^ and tumor-associated stroma have been implicated in cancer progression and patient survival^26^.

We present a digital pathology-based pipeline that is able to prognosticate DFS in early stage ER+ IBC by automatically quantifying the Collagen Fiber Orientation Disorder in Tumor-associated Stroma (CFOD-TS) directly from digitized routine H&E image. Our computational pathology-derived biomarker could be more readily transitioned into clinical practice by taking advantage of the rapid increase in the availability of WSI scanning^27^, compared to the SHG/LSM microscopy-based biomarkers. Specifically, the automated pipeline consists of image processing techniques combined with a machine learning model to segment tumor-associated stroma on H&E slide images, from which we subsequently detected the collagen fiber orientations. Subsequently, we utilized entropy theory^28^ to calculate CFOD-TS, in order to quantitatively characterize the degree of disorder of the collagen fiber orientations at the tumor leading edge as well as across the entire tumor region. Following that, 78 patients (number of DFS event = 34) with early stage ER+ IBC identified from The Cancer Genome Atlas Breast Invasive Carcinoma (TCGA BRCA) were used as the training set (S_t_) to construct a Cox proportional regression model (M_CFOD-TS_) to predict DFS based on the CFOD-TS features. 219 breast cancer patients (number of DFS event = 64) from the completed Eastern Cooperative Oncology Group 2197 (ECOG 2197) were employed (S_v_) for validating M_CFOD-TS_. ECOG 2197^29^ is a randomized phase III clinical trial to compare the effectiveness of two combination chemotherapy regimens in treating early stage IBC (no significant difference observed in cancer outcome between the two regimens)^29^. The prognostic significance of M_CFOD-TS_ was also validated on intermediate (indeterminate) Odx category as well as the subgroup of lymph node negative (LN−) and lymph node positive (LN+) patients in S_v_. Additionally, we explored the relationship between CFOD-TS characterized phenotype and the underlying genotype utilizing the genomic data in S_t_.

## Results

### Patient characteristics

Clinicopathological variables for S_t_ and S_v_ are provided in Table 1. In S_t_, 34 patients (45%) had an event (recurrence or death) with median DFS being 1379 days versus 65 patients (30%) with an event and median DFS of 3517 days in S_v_. All patients in S_v_ received adjuvant chemotherapy while treatment related information was only available for 6 patients in S_t_, with 4 of them receiving adjuvant chemotherapy. Other than tumor size and age, no clinicopathological variables were found to be significantly different between S_t_ and S_v_. S_v_ was considered a rigorous validation set of M_CFOD-TS_ given that the relative homogeneity in terms of treatment regimen (all patients received chemotherapy) and cancer clinical characteristics enabled the outcome to be more reflective of the inherent aggressiveness of breast cancer.

**Table 1:** Summary of clinicopathological variables and Odx risk categories in S_t_ and S_v_.

Clinical variable	S_t_: *N* (%)	S_v_: *N* (%)
*Lymph node*		
node−	23 (29%)	116 (53%)
node+	36 (46%)	103 (47%)
unknown	19 (25%)	0 (0%)
*PR*		
pr+	66 (85%)	185 (84%)
pr−	12 (15%)	34 (16%)
*HER2*		
her2+	15 (19%)	40 (18%)
her2−	33 (42%)	88 (40%)
unknown	30 (39%)	91 (42%)
*Tumor grade*	Not available	
grade 1		32 (15%)
grade 2		107 (49%)
grade 3		56 (26%)
unknown		24 (10%)
*Tumor size*		
≤2 cm	20 (26%)	95 (43%)
>2 cm	58 (74%)	122 (56%)
unknown	0 (0%)	2 (1%)
*Age*		
≥50 years	21 (27%)	89 (41%)
< 50 years	56 (72%)	130 (59%)
unknown	1 (1%)	0 (0%)
*Oncotype Dx*	Not available	
Low		14 (6%)
Intermediate		28 (13%)
High		17 (8%)
Unknown		160 (73%)
*Race*		
Caucasian	61 (78%)	198 (90%)
Non-Caucasian	12 (15%)	21 (10%)
Unknown	5 (7%)	0 (0%)
*Histology type*		Not available
Ductal invasive carcinoma	60 (77%)	
Lobular invasive carcinoma	11 (14%)	
Unknown	7 (9%)	

### Experiment 1: CFOD-TS prognostic of DFS in early stage ER+ breast cancer

**Fig. 1:**
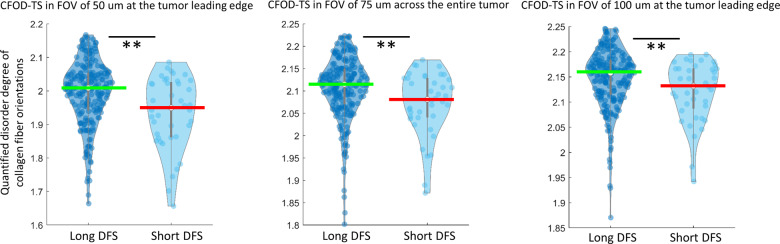
Top CFOD-TS features distribution. Distribution of top three CFOD-TS features between patients with long DFS (DFS ≥ 5 years) and short DFS (with an event & DFS < 5 years) in S_t+v_ (S_t_ + S_v_) with each point representing one single patient, including CFOD-TS features derived from FOV (left to right) in 50 um at tumor leading edge; in 75 μm across the whole tumor and 100 μm at the tumor leading edge.

A total of 8 CFOD-TS features were identified in the LASSO regularized Cox regression modeling with DFS on S_t_ (*N* = 78) and integrated into M_CFOD-TS_. The number of top features was determined as approximately 10% of the patient number in S_t_. All 8 CFOD-TS features were found to be significantly (*p* < 0.05 from WRST) lower in patients with short DFS (with an event and DFS < 5 years) compared to those with long DFS (DFS > = 5 years) in S_t+v_ (S_t_ + S_v_). The eight identified features consisted of the following: CFOD-TS in the FOV of 50, 100, 250 μm at the tumor leading edge and CFOD-TS in FOV of 75, 125, 150, 200, 225 μm across the whole tumor region. Notably, among the eight identified top features, CFOD-TS features derived from relatively small FOVs were found to be more distinguishable (smaller *p* value from WRST) between short and long DFS patient groups (Fig. 1).

**Fig. 2:**
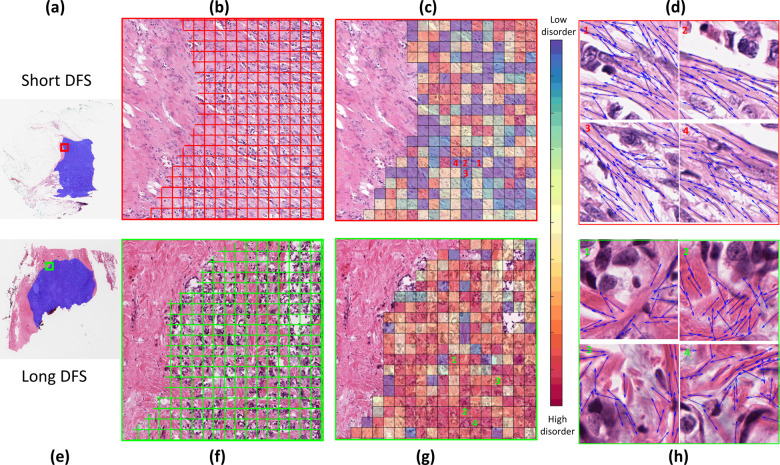
Representative WSI of breast cancer tissue for patients with short DFS (top row) versus long DFS (bottom row). The first column (**a**, **e**) showed the original H&E stained WSIs with tumor mask (blue) overlaid. The second column (**b**, **e**) showed the magnified tumor region at the leading edge, which was compartmentalized into a grid of smaller tumor neighborhoods of 50 um × 50 um. The third column (**c**, **g**) illustrates the quantified CFOD-TS in each individual tumor neighborhood within the tumor region in (**b**) and (**f**), where a warm color illustrates a higher feature value (higher disorder degree of fiber orientations); The fourth column (**d**, **h**) illustrates the organization of the collagen fiber orientations (the orientations indicated by the blue arrows) in the tumor-associated stroma in representative tumor neighborhoods (position of each tumor neighborhood was indicated by the corresponding number on (**c**) and (**g**)). The patient with short DFS was characterized by a more aligned and ordered collagen fiber organization, while the patient with long DFS presented a higher degree of disorganization of collagen fiber orientation.

Figure 2 shows the visualization of collagen fiber organization at the tumor edge for a short and long DFS patient respectively. An aligned collagen fiber organization could be observed in the high-risk patient based on both feature map (Fig. 2c, g) and closer visual inspection of the cancer tissues (Fig. 2d, h).

**Fig. 3:**
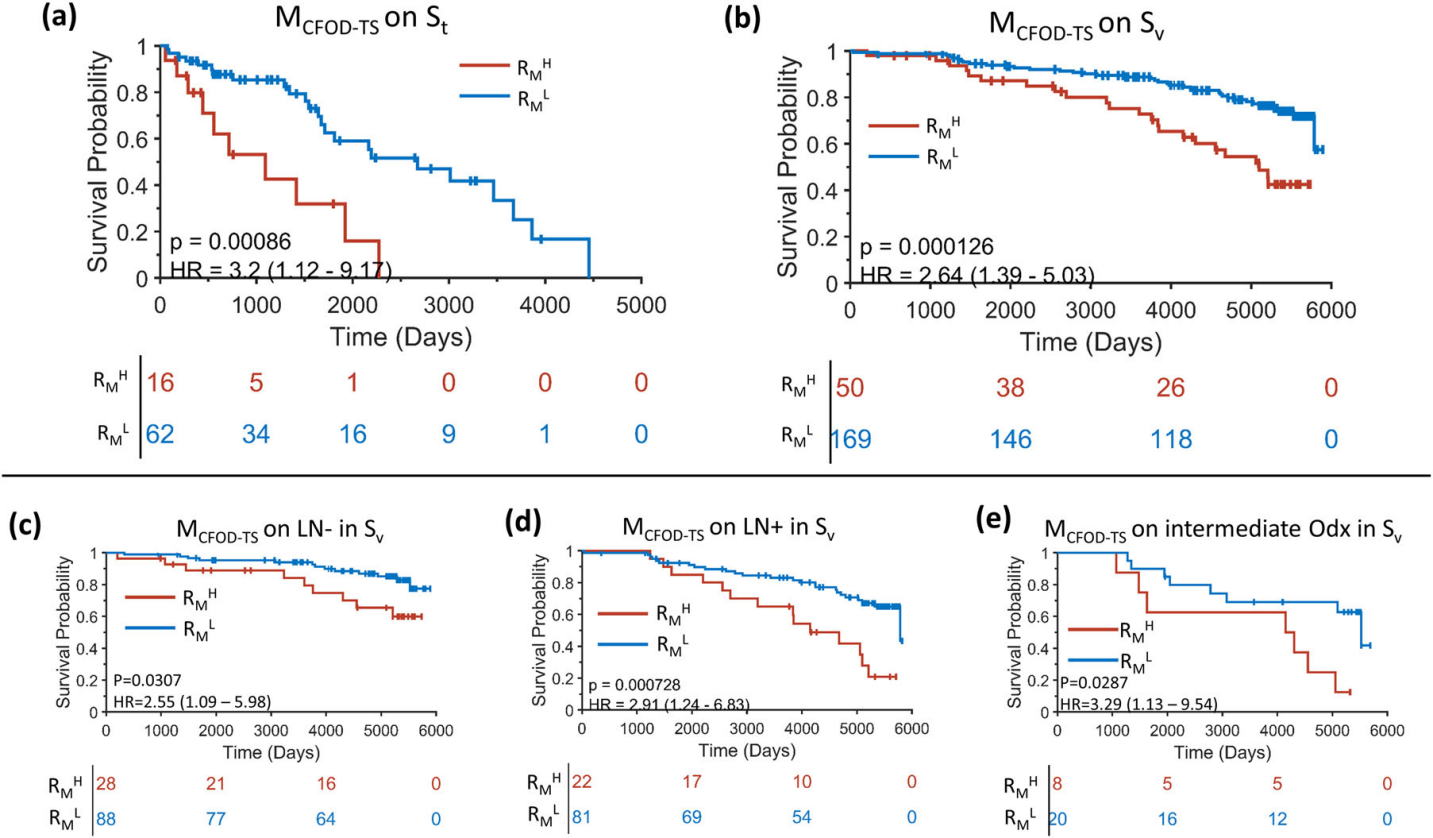
Prognostic performance of M_CFOD-TS_ on S_t_ and S_v_. KM curve estimates of DFS on R_M_^L^ versus R_M_^H^ on S_t_ (**a**), S_v_ (**b**) and subgroups in S_v_ (**c**–**e**) including subgroup of lymph node negative (**c**), lymph node positive (**d**) and intermediate Odx risk category (**e**) patients. The number of patients at risk at each time point is specified below the KM curves.

A risk category R_M_ was predicted from M_CFOD-TS_ by dichotomizing the sum of a linear combination of the identified eight top features as described in the “Statistical analysis” section. The KM survival curves of DFS between R_M_^H^ and R_M_^L^ groups are plotted for both S_t_ (Fig. 3a) and S_v_ (Fig. 3b). A significantly favorable outcome in R_M_^L^ compared to R_M_^H^ was observed with HR = 3.2 (95% CI = 1.12–9.17, *p* = 0.00086) on S_t_ and HR = 2.64 (95% CI = 1.39–5.03, *p* = 0.000126) on S_v_ using a log-rank test. In the subgroup survival analysis on S_v_ (Fig. 3c, d), M_CFOD-TS_ was also found to be prognostic in the subgroup of the LN− patients with HR = 2.55 (95% CI = 1.09–5.98, *p* = 0.031) (Fig. 3c) and LN+ patients with HR = 2.91 (95% CI = 1.24–6.83, *p* = 0.000728) (Fig. 3d). Furthermore, M_CFOD-TS_ was found to significantly risk stratify intermediate Odx risk category patients (HR = 3.29, 95% CI = 1.13–9.54, *p* = 0.0287) (Fig. 3e).

### Experiment 2: Univariate and multivariate analysis

Table 2 illustrates the results of univariate and multivariate analysis of clinicopathological variables/Odx risk category/R_M_ for DFS. In the univariate analysis setting, other than lymph node status in S_v_, neither clinicopathological variables nor Odx risk category was identified as being significantly prognostic of DFS on S_t_ and S_v_. Patients in R_M_^H^ had significantly worse DFS (HR = 3.49, 95% CI = 1.6–7.6, *p* = 0.001 on S_t_; HR = 2.67, 95% CI = 1.58–4.5, *p* = 2.28e−04 on S_v_) compared to those in R_M_^L^. In the multivariate analysis controlling the effects of the clinicopathological variables, R_M_ was found independently prognostic of DFS with HR = 3.45 (95% CI = 1.03–12, *p* = 0.04) on S_t_ and HR = 3.82 (95% CI = 2.14–6.8, *p* = 5.79e−06) on S_v_. The prognostic significance of lymph node status in S_v_ still held after adjusting the impact of other clinicopathological variables.

**Table 2:** Univariate and Multivariate Cox proportional hazards analysis for DFS on R_M_/clinicopathological variables/Odx risk category on S_t_ and S_t_.

Clinicopathological Variables	TCGA BRCA Hazard Ratio (p, 95% Confidential Interval), patient number	ECOG 2197 Hazard Ratio (p, 95% Confidential Interval), patient number
Univariate analysis		
Lymph node (node+ vs. node−)	HR = 0.85 (*p* = 0.69, CI = 0.37–1.9), *N* = 59	HR = 2.15 (*p* = 0.0042, CI = 1.27–3.6), *N* = 219
PR (PR+ vs. PR−)	HR = 1.17 (*p* = 0.73, CI = 0.48–2.9), *N* = 78	HR = 0.71 (*p* = 0.39, CI = 0.32–1.6), *N* = 219
HER2 (HER2+ vs. HER2−)	HR = 0.95 (*p* = 0.93, CI = 0.33–2.7), *N* = 48	HR = 1.25 (*p* = 0.57, CI = 0.58–2.7), *N* = 128
Tumor grade (grade 3 vs. grade 2 vs. grade 1)	Variable not available	HR = 0.88 (*p* = 0.54, CI = 0.59–1.3), *N* = 195
Tumor size (≥2 cm vs. <2 cm)	HR = 1.27 (*p* = 0.6, CI = 0.52–3.1), *N* = 78	HR = 1.5 (*p* = 0.13, CI = 0.88–2.5), *N* = 217
Age (≥50 years vs. <50 years)	HR = 1.06 (*p* = 0.87, CI = 0.51–2.2), *N* = 77	HR = 1.17 (*p* = 0.54, CI = 0.7–2), *N* = 219
Oncotype Dx category (high vs. intermediate vs. low)	Variable not available	HR = 1.61 (*p* = 0.09, CI = 0.93–2.8), *N* = 59
Race (non-caucasian vs. caucasian)	HR = 1.14 (*p* = 0.79, CI = 0.43–3), *N* = 73	HR = 0.71 (*p* = 0.5, CI = 0.26–1.9), *N* = 219
Histology type (lobular invasive carcinoma vs. ductal invasive carcinoma)	HR = 0.21 (*p* = 0.13, CI = 0.03–1.6), *N* = 71	Variable not available
Model-derived risk category (RM^H^ vs. RM^L^)	HR = 3.49 (*p* = 0.001, CI = 1.6–7.6), *N* = 78	HR = 2.67 (*p* = 2.28e−04, CI = 1.58–4.5), *N* = 219
Multivariate analysis		
Clinicopathological Variables	TCGA BRCA (*N* = 47)	ECOG 2197 (*N* = 193)
Lymph node (node+ vs. node−)	HR = 0.35 (*p* = 0.1, CI = 0.1–1.2)	HR = 2.75 (*p* = 0.001, CI = 1.49–5.1)
PR (PR+ vs. PR−)	HR = 9.26 (*p* = 0.01, CI = 1.72–50)	HR = 0.82 (*p* = 0.63, CI = 0.36–1.9)
Tumor grade (grade 3 vs. grade 2 vs. grade 1)	Variable not available	HR = 1.02 (*p* = 0.93, CI = 0.66–1.6)
Tumor size (≥2 cm vs. <2 cm)	HR = 1.01 (*p* = 0.99, CI = 0.28–3.6)	HR = 1.57 (*p* = 0.12, CI = 0.89–2.8)
Age (≥50 years vs. <50 years)	HR = 1.04 (*p* = 0.95, CI = 0.35–3)	HR = 1.02 (*p* = 0.93, CI = 0.58–1.8)
Race (non-caucasian vs. caucasian)	HR = 1.75 (*p* = 0.48, CI = 0.37–8.3)	HR = 1.51 (*p* = 0.44, CI = 0.53–4.3)
Histology type (lobular invasive carcinoma vs. ductal invasive carcinoma)	HR = 0 (*p* = 0.99, CI = 0~ Inf)	Variable not available
Model-derived risk category (R_M_^H^ vs. R_M_^L^)	HR = 3.45 (*p* = 0.04, CI = 1.03–12)	HR = 3.82 (p = 5.79e–06, CI = 2.14–6.8)

The latter subgroup for each variable (e.g. node -) was used as the reference category for HR calculation

### Experiment 3: Identification of associated biological pathways with CFOD-TS features and enrichment analysis

Out of the 60488 annotated genes for TCGA BRCA, 1062 genes (Supplementary List 1) were significantly differentially expressed between patients assigned to the R_M_^H^ and R_M_^L^. Among the GO identified pathways with the 1062 genes as the input, ten molecular pathways (Supplementary Table 2) were considered as regulating the potential underpinning biological process of the CFOD-TS defined phenotype. Six of the pathways were directly related to extracellular or fiber organization while four pathways were chosen considering their biological significance in regulating cell development and cell division. None of the enrichment scores calculated by ssGSEA for the six pathways regarding ECM organization was significantly associated with M_CFOD-TS_ derived risk scores. However, among the four identified pathways describing the cell development and division process, Cell Cycle Arrest (CCA) and Regulation of Cell Cycle (RCC) were identified to be significantly associated with M_CFOD-TS_ risk scores, with the Pearson correlation coefficients respectively being −0.35 (*p* = 0.0017) and −0.31 (*p* = 0.0069). CCA^30^ is a checkpoint in the cell cycle before proceeding to duplication and division. CCA was found to be overexpressed in M_CFOD-TS_^L^ patients, suggesting that the mechanism facilitating DNA repair or cell cycle progression was differentially regulated in low risk compared to high risk patients. Similarly, RCC^31^ was also found to be negatively associated with M_CFOD-TS_ derived risk scores, suggesting a more dysregulated cell division process in R_M_^H^ patients.

## Discussion

Increasing evidence has emerged to^6,7,11,17^ demonstrate the critical role of collagen fiber organization in breast cancer progression. While multiple studies^11,17^ have been conducted to identify prognostic biomarkers for invasive breast cancer (IBC) based on the quantitative measurement of collagen fiber organization, these studies have typically involved the use of advanced microscopy imaging technologies like Second Harmonic Generation (SHG) or Laser Scanning Microscopy (LSM) system. Those technologies, however, are far from translation into the routine clinical pathology workflow. With the advent of digital pathology, there has been substantial research interest in investigating computationally extracted quantitative histomorphometric attributes such as shape and spatial arrangement of cancer nuclei and immune cells on H&E images for breast cancer prognosis^21,22,26,32^. However, to the best of our knowledge, no study has attempted to quantitatively measure collagen fiber organization directly on standard H&E slide images and relate these measurements to breast cancer outcome.

The objective of this study is to quantitatively measure the organization of collagen fibers from routine H&E slide images alone and investigate its association with the outcome (DFS) for early stage ER+ IBC. Utilizing a set of imaging processing techniques and a state-of-the-art deep learning model, we were able to quantitatively characterize Collagen Fiber Orientation Disorder in Tumor-associated Stroma (CFOD-TS) solely with routine H&E stained images, without the need of special collagen staining or advanced imaging and microscopy techniques. Following the CFOD-TS feature extraction, we constructed a Cox regression model (M_CFOD-TS_) with CFOD-TS features to predict DFS, with a continuous risk score as the output. M_CFOD-TS_ was validated to be prognostic on an independent, archived clinical trial dataset (ECOG 2197) independent of clinicopathologic variables e.g. tumor grade, tumor size. M_CFOD-TS_ was also found to be prognostic on the patients in the subgroups of LN−, LN+ and intermediate Odx risk category. Additionally, the molecular pathways relevant to cell cycle regulation were identified as associated with the collagen fiber organization phenotype. We found the aligned and ordered organization of collagen fibers was indicative of a worse prognosis in terms of DFS for early stage ER+ IBC.

Oncotype Dx (Odx) is a multi-gene test which is prognostic and predictive of benefit of adjuvant chemotherapy for early stage IBC^5^. While the high and low Odx risk categories have very clear prognostic and predictive significance, the intermediate Odx risk category is more ambiguous and results in challenges to the physicians in interpreting results for patients assigned to this category, especially with making appropriate treatment decisions^4^. Our model was able to risk stratify patients in the intermediate Odx risk category with HR = 3.29 (95% CI = 1.13–9.54, *p* = 0.0287). Most of the patients (20/28) in intermediate Odx were identified as low risk by M_CFOD-TS_ and among the 8 patients in R_M_^H^ group, 7 patients recurred or died along the course of follow-up. While Odx test has been validated to be prognostic on ECOG 2197^5^, Odx test did not show prognostic significance in the univariate analysis for DFS (Table 2) in our study. Potential reasons for this could include (1) instead of using recurrence rate within five years, we used DFS as the end point, (2) we only had access to a subset of patients in ECOG 2197, and (3) we employed the updated risk thresholds as recommended by the TAILORx prospective study^33^ instead of the traditional categorization^4^ to divide the high/intermediate/low Odx category.

Quantitative measurement (Fig. 1) and qualitative visualization (Fig. 2) of the CFOD-TS features revealed the patients with short DFS tend to have a more aligned fiber organization, which appears to be in line with previous studies^8,12,34^. For example, multiple investigators^15,16,35^ have shown that in mice model, cancer cells preferentially invaded along aligned collagen fibers into the adjacent stroma, and focal sites of breast cancer cell micro-invasion often co-existed with stiff aligned collagen fibers. Also, Bredfeldt et al^17^. and Conklin et al^11^. found that the presence of straightened, aligned collagen fibers at a local tumor boundary observed on SHG images was indicator of poor prognosis for IBC patients. One of the advantages of our present pipeline is that we expanded the samples from tumor cores that previous studies^11,17^ have utilized in the collagen fiber organization analysis to WSIs, which harbor more comprehensive tumor characteristic information. A number of hypotheses^14,34,36,37^ have been proposed to demonstrate the mechanism behind how the aligned collagen fibers facilitated tumor migration and invasion. In the aspect of physical mechanism, given that the cancer cells have been observed to migrate rapidly along aligned collagen fibers, it has been assumed that collagen realignment could eliminate the physical barrier impeding the moving cells^16^ and generate tube-like trails of least resistance to promote cell movement^14,34^. For example, Riching et al^34^. found that by performing a microchannel migration assay to track the tumor cells traveling in a 3D collagen gel, the tumor cells were able to travel greater distances in aligned collagen fiber gels compared to the randomly organized collagen gels within the same time period. This observation suggests that the efficiency of tumor migration is enhanced in aligned fibers, possibly by limiting protrusions along the fibers. Some researchers^38,39^ also identified molecular involvement in the facilitated tumor cell movement along the aligned collagen fibers. For example, Wang et al.^38^ found that the cancer cells on highly aligned ECM possessed organized Focal Adhesions (FAs). Highly aligned FAs and associated F-actin stress fibers in turn resulted in the localization of active Rac1 which further stabilized the cell protrusions along the direction of ECM alignment.

The CFOD-TS feature calculated from a small FOV was found to be more discriminating between patients with long and short DFS compared to the features derived from a larger FOV. A possible explanation for this is that the fibers in a small FOV are more likely to constitute a micro-environment, functioning as a unit. The fibers mutually and directly interact with the tumor, and further facilitate tumor migration through the alignment in the orientations, which in turn is quantified as low CFOD-TS. However, a large FOV (big tumor neighborhood) could actually comprise multiple independent fiber-composed micro-environments. In this case, the calculated CFOD-TS not only reflects the fiber orientation alignment within each individual micro-environment as in the case of a small FOV, but also captures the relative orientation alignment among the multiple micro-environments. However, considering that the multiple micro-environments within a larger FOV affect the tumor behavior independently, the corresponding calculated CFOD-TS from the large FOV might end up being less prognostic. Additional experiments were also performed to allow for more intuition with respect to the extracted CFOD-TS features. A high correlation between CFOD-TS at the tumor leading edge and across the whole tumor region (Supplementary Fig. 2) indicated the realignment of collagen fibers occurs simultaneously across the entire tumor and is not significantly affected by the spatial location of the fibers. A higher FOV yielded a higher averaged degree and a lower variation in collagen fiber orientation disorganization across the whole tumor region (Supplementary Fig. 3). Considering that the calculation of the disorganization feature within a large FOV takes into account fibers from across a large tumor neighborhood, the results could indicate that fiber orientations were less likely to uniformly align at a large scale (across a large tumor neighborhood). Instead, the orientation alignment was often limited to only those fibers that were adjacently located within the same small tumor neighborhood.

A number of studies have been conducted to interrogate the prognostic value of computerized tumor morphometric features on H&E slide images for IBC, either based on quantitative handcraft histomorphometric features^21,23^ or using deep learning based neural networks^40^. The handcraft morphological attributes have mostly been focused on nuclear histomorphometric features in tumor epithelium or tumor stroma. For example, on a cohort of 378 early stage IBC patients from the same clinical trial (ECOG 2197) as in our study, Verma et al^41^. built a prognostic model to classify patients into low- versus high-risk groups for recurrence by leveraging the nuclear morphometric signatures from H&E slide images, with HR = 2.41 (95% CI = 1.21–4.79, *p* = 0.01). In addition, they found that by combining the nuclear-morphology-based prognostic model with the Odx risk category, 20% more patients in low risk could be correctly identified compared to using Odx test alone. Similarly, Lu et al.^21^ found that nuclear texture heterogeneity as well as the nuclear orientation disorder were associated with overall survival for early stage IBC patients. Apart from the handcraft features, deep learning based neural network models have also been used to directly prognosticate cancer outcome based on pathology slide images. Wulczyn et al.^40^ developed and trained a deep learning system consisting of a weakly supervised approach and survival loss function using H&E slide images in TCGA. The risk scores generated from the deep learning classifier were validated as being significantly associated with DFS in IBC, after adjusting for the impact of age and stage. However, different from previous studies in computerized histomorphology analysis, our study quantitatively measured collagen fiber orientation organization on H&E slide images and associated with the breast cancer outcome. Our study demonstrated that CFOD-TS, which is not typically examined to assess disease prognosis in pathological practice, contains potential valuable prognostic information that could be captured by visual inspection on H&E slides.

In this work, we also explored the molecular underpinning of CFOD-TS on TCGA BRCA dataset with available mRNA sequencing data. Two biological pathways including Cell Cycle Arrest (CCA) and Regulation of Cell Cycle (RCC) were identified as being significantly associated with the M_CFOD-TS_ derived risk scores. CCA^30^ is often used by cells to facilitate DNA repair and cell cycle progression. A down-regulation of CCA pathway was found in the high-risk patients identified by M_CFOD-TS_, which indicated a dysregulated tumor division process. Similarly, RCC^31^ was also found to be down-regulated among the M_CFOD-TS_ identified high-risk patients, which further suggested a defected regulation of division or duplication process of cancer cells. The mechanism of ECM interacting with the molecular processes within tumor cells has been demonstrated in previous studies^42,43^. Mouw et al. found that the increased ECM stiffness led to reduced expression of the tumor suppressor phosphatase^43^; meanwhile, the increase in ECM stiffness was also identified as one of the causes of collagen fiber realignment^42^. Those findings provide an insight into the possible molecular mechanism behind the association between the suppressed cell cycle regulation and model-derived collagen orientation risk score developed in our study.

We do acknowledge that the study did have its limitations. Since we did not have access to specialized collagen-specific stained slide images, we were unable to definitively validate the segmentation performance of our algorithm for identifying collagen fibers on the H&E images. However, we were still able to accurately and quantitatively measure the collagen fiber orientation disorder by capturing the directional pattern of the collagen fibers on H&E slide images (accuracy was validated by visual inspection from pathologists) without requiring stringent discernment on each individual collagen fiber. Another limitation pertaining to this study is the relatively small data size due to the restrictive patient inclusion criterion to retain the homogenous nature of the cohorts. The purpose of narrowing the scope down to relative homogenous datasets in terms of tumor stage is to eliminate the co-effect of the tumor stage on the CFOD-TS aside from the effect from inherent tumor aggressiveness. Also, unlike the Odx test, which was prospectively validated in both prognostic significance and treatment benefit prediction, our study was retrospectively validated on a single clinical trial dataset and only validated in terms of prognostic ability. Future work will entail validating the pathology image-based prognostic pipeline in larger, additional independent pan-stage cohorts, and also in terms of its predictive benefit for adjuvant chemotherapy. Additionally, we will expand our validation into the pathology images scanned under different parameters (e.g. microns-per-pixel) and for other molecular subtypes, such as triple negative breast cancer. Furthermore, we will look to expand our CFOD-TS analysis into tumor adjacent non-oncologic tissue in future studies. Specifically, we will use the non-oncologic tissue as a reference to investigate the disruption of CFOD-TS by the tumor cells, and study how the disruption in the organization is associated with the tumor aggressiveness.

In summary, this study quantitatively measured the collagen fiber organization in breast tumor-associated stroma solely using routine H&E stained images and demonstrated its prognostic significance in terms of DFS for early stage ER+ IBC.

## Methods

### Dataset

Two cohorts were used in this study: TCGA BRCA (S_t_: *N* = 78) and ECOG 2197 (S_v_: *N* = 219). A flow diagram describing the inclusion and exclusion criterions on each of the datasets is provided in Supplementary Fig. 1). The primary inclusion criteria for this study were ER+, early stage (LN+/high risk LN−) breast cancers, with associated DFS and digital slide presenting a reasonable amount of IBC tissue for subsequent image analysis.

S_t_ was employed as a training set for feature discovery and model construction. The dataset included 78 patients with the digital slides of Formalin-Fixed Paraffin-Embedded (FFPE) IBC tissue (×40 magnification (mag): ~0.25 μm (um)/pixel). The corresponding clinicopathological and outcome data were downloaded from Genomic Data Commons (GDC) data portal. In order to keep relative consistency with respect to tumor stage with S_v_, only the patients with Stage II (Tumor, Node, Metastasis staging system) or high risk (tumor size > = 1 cm) LN− tumors were recruited into this cohort. All the patients with an DFS event (recurrence or death) meeting the inclusion criteria, matched with a set of censored patients (no DFS event) from TCGA BRCA were used to constitute S_t_.

S_v_ was used as an independent validation set to evaluate the model performance. The ECOG 2197 trial was a prospective, randomized, clinical trial from 1998 to 2007 that recruited patients with IBC (1–3 positive LN/LN− with tumor size > = 1 cm) to compare the patient’s outcome under two different chemotherapy regimens^29^. 219 patients with ER+ IBC were selected to comprise S_v_ after the inclusion criteria applied on the 256 patients in ECOG 2197, for whom we had access to both the corresponding de-identified slide images and relevant clinical information. The access to the ECOG dataset involved 2-year process including a proposal review first through ECOG and subsequently through Cancer Therapy Evaluation Program (CTEP) in National Cancer Institute (NCI). In ECOG 2197, the effects of treatment and clinical characteristics of cancers on the outcome were relatively controlled thus enabling the outcome to be more reflective of the nature aggressiveness of breast cancer and making S_v_ an ideal validation set. All the FFPE tissue slides were scanned and digitized using a Philips scanner at 40x mag. Clinicopathological and outcome information were obtained from retrospective chart review.

The study conformed to HIPAA guidelines was approved by the Institutional Review Board (IRB) at University Hospitals Cleveland Medical Center. IRB No 02-13-42C. The need for written consent from participants was waived due to the use of de-identified retrospective data.

### Automated detection of collagen fiber orientations in tumor-associated stroma on H&E slide images

Following the acquisition of the digital WSIs, HistoQC^44^, a quality control tool for digital pathology slides was employed to identify the useful tumor tissue by excluding fat tissues and regions with artifacts e.g. tissue folding, pen marking, and blurriness.

**Fig. 4:**
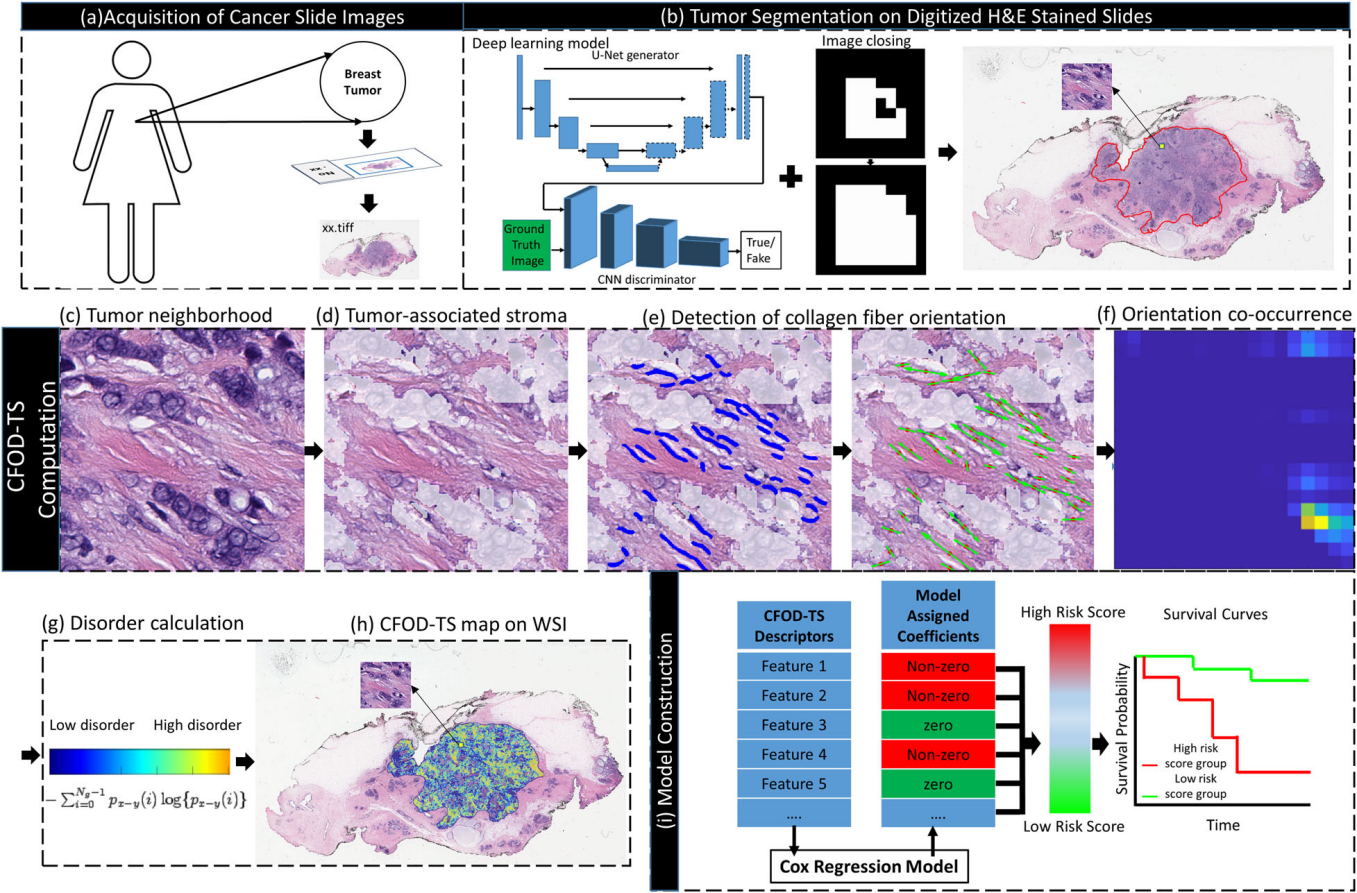
Flowchart of experimental design. The flowchart illustrates the complete experimental design. **a** Digitized WSIs were collected from two cohorts, in which TCGA BRCA was the training set (S_t_), and ECOG 2197 was the independent validation set (S_v_). **b** A deep learning model combined with post image processing techniques were utilized to segment the tumor region on WSI. **c**–**h** Quantitative features of Collagen Fiber Orientation Disorder in Tumor-associated Stroma were calculated. A tumor neighborhood in a specified radius was defined (**c**). A deep learning model was applied to the tumor neighborhood to segment the stroma area, which was defined as tumor-associated stroma (**d**). The collagen fiber orientations in tumor-associated stroma were captured using a linear structure detection-based model (**e**). An orientation co-occurrence matrix was subsequently constructed with a brighter co-occurrence value in the on-diagonal cells suggesting higher co-occurrence of collagen fibers of the same orientation (**f**). The feature quantifying the degree of disorder of collagen fiber orientations was then calculated from the matrix (**g**). The tumor neighborhood slid across the whole tumor region to generate a CFOD-TS feature map (**h**). The average value of the feature map was calculated at the tumor leading edge as well as across the whole tumor. **i** Following extraction of a set of CFOD-TS features, A Cox regression model (M_CFOD-TS_) was constructed on S_t_ to discover top features by assigning a corresponding coefficient to each of the features, based on which a continuous risk score was generated. Kaplan–Meier (KM) survival analysis was performed on the patients with M_CFOD-TS_ derived low risk scores versus high risk scores on both S_t_ and S_v_.

A conditional Generative Adversarial Network (cGAN) model was implemented on the useful tumor tissues on the digitized WSIs identified by HistoQC. The cGAN model is an extension of GAN with both the generator and discriminator being conditioned on auxiliary information^45^. The cGAN model (deep learning model in Fig. 4b) consisted of a standard U-Net structure as the generator and a multi-layer convolutional network as the discriminator. The model was trained using 1286 images with a pixel size of 512 × 512 from a total of 576 H&E stained breast cancer pathological images provided by the Netherlands Cancer Institute^46^ and Vancouver General Hospital^47^. All images had annotations of epithelial and stromal regions provided by pathologists under 20x mag. Following acquisition of cGAN generated epithelial and stroma mask of the breast cancer tissues on WSIs, a set of morphological operations such as image dilation and holes-filling were employed to merge the individual epithelium patches thus automatically generating a tumor mask (Fig. 4b). The stroma region inside the merged tumor mask was defined as tumor-associated stroma. The tumor masks were overlaid on the original tissue images and visually inspected by a pathologist, and, if deemed necessary, manually edited to ensure that the tumor region was accurately delineated.

Collagen fibers are composed of fibril bundles that are linear arrays of type I collagen molecules^48^, thus usually presenting with a linear phenotype on H&E slide images of breast cancer. A derivative-of-Gaussian (DtG) based model described in Griffin et al.’s^49^ work was used to capture the fiber orientation by detecting the linear structures in tumor-associated stroma on H&E slide images. Specifically, the DtG based model classified each pixel on the slide image as being within one of seven image structures (Flat, Slope, dark Blob, light Blob, dark Line, light Line and Saddle). By retrieving the image pixels identified as belonging to a linear fiber (dark Line structure), we were able to capture the directional pattern of the collagen fibers (Fig. 4e). The accuracy of the collagen fiber orientation detection was visually assessed by an attending pathologist and a resident pathologist independently on 100 tumor tiles randomly cropped respectively from 100 sample WSIs. The tumor tiles were marked with arrows pointing to the fiber orientations captured by our approach. The two pathologists checked the tiles and assigned it to one of four categories (excellent, good, fair and poor) to describe the accuracy of the captured collagen fiber orientations. 94% of tiles were ranked as excellent/good, 6% as fair/poor by the resident pathologist. The attending pathologist ranked 77% of the tiles as excellent/good, 22% as fair and 1% as poor.

### Extraction of collagen fiber orientation disorder in tumor-associated Stroma (CFOD-TS) features

We partitioned the WSI into an array of local tumor neighborhoods where the respective disorder degree of fiber orientations was calculated. Specifically within each individual tumor neighborhood, the direction of each detected linear structure (*V*_*i*_) representative of collagen fiber orientation was denoted as $\varTheta({V}_i)$. The $\varTheta({V}_i)$ was the angle between the horizontal axis and the major axis of a ellipse that has the same second-moments as *V*_*i*_, ranging between 0 degree and 180 degree. The orientations were then discretized to angular bins from 0 to 17. An orientation co-occurrence matrix (Fig. 4f) was constructed based on the set of $\varTheta({V}_i)$ within the tumor neighborhood (Fig. 4c), with each row/column corresponding to an angular bin. The value for each cell in the matrix was calculated based on the co-occurrence frequency of the two orientations corresponding to the row and column. The equation describing how the orientation co-occurrence matrix was calculated is included in Supplementary Formula 1.

The quantitative measurement of Collagen Fiber Orientation Disorder in Tumor associated Stroma (CFOD-TS) was subsequently calculated from the orientation co-occurrence matrix based on entropy theory^28^ (Fig. 4g). The entropy measurement reflects the level of "information" or "uncertainty" in the collagen fiber’s orientation in the neighborhood of the tumor. The equation for orientation disorder calculation is in Supplementary Formula 2. Based on the tiles ranked as fair/poor by the pathologists, the collagen fiber orientation detection algorithm yielded a sub-optimal performance when the tile was dominated by epithelium with a small portion of stroma present as well as when the linear phenotype of the collagen fiber was not present in the tumor-associated stroma of the tile. In order to address these issues, if the stroma ratio (ratio of stromal area to tumor neighborhood) or the number of detected fiber orientations for a tumor neighborhood was lower than a pre-defined threshold, the neighborhood was considered as containing too sparse fiber-enriched stroma content to derive an effective CFOD-TS measurement and thus was disregarded for the subsequent disorganization calculation. Following the calculation of CFOD-TS for each individual compartmentalized tumor neighborhood, an average value of the features derived from all the tumor neighborhoods was calculated as the patient-level feature. The size of tumor neighborhood (FOV) needs to be defined, but no prior knowledge exists as to what the optimal FOV is for collagen fiber disorganization measurement. Therefore, we applied nine different FOVs ranging from 50 to 250 um for feature extraction. Apart from the measurements from the compartmentalized local tumor neighborhoods across the whole tumor region in the WSI, we also assessed the CFOD-TS exclusively in the tumor neighborhoods at the tumor leading edge (within 500 μm of the tumor mask periphery), which is roughly the border of tumor cells aggressing and migrating into adjacent normal stromal region. Consequently, we finally obtained a total of 18 features (Supplementary Table 1).

### Statistical analysis

DFS was defined as the time interval between the date of diagnosis/randomization to the date of recurrence, death and was censored at the date of last follow-up for those patients alive without recurrence. Cox Proportional Hazard Model^50^, henceforth referred to as Cox regression model, relates the time that passes until some event occurs, to multiple covariates. We regularized the Cox regression model by a Least Absolute Shrinkage and Selection Operator (LASSO) penalty function to identify important predictors of DFS. We constructed a Cox regression model named M_CFOD-TS_ on S_t_ between CFOD-TS features and DFS. A corresponding coefficient was assigned to each of the features in the final model. A continuous risk score was then calculated from a linear combination of the top features weighted by the corresponding coefficients for each individual patient (Fig. 4d). The model was further validated on S_v_ by applying the same set of feature coefficients.

Kaplan–Meier (KM) survival analysis was used to analyze the difference of the expected duration of time until an event happens between two defined categories. We converted the continuous risk scores calculated from M_CFOD-TS_ into a binary high (R_M_^H^: risk score > threshold) vs. low (R_M_^L^: risk score< = threshold) risk categories. The threshold was selected so as to give the most significant prognosis on S_t_. KM curves were generated to compare the DFS between R_M_^H^ and R_M_^L^ groups, with the difference assessed by the log-rank test on both S_t_ and S_v_. Additionally, we performed a dedicated survival analysis for different subgroups in S_v_. KM curves were plotted respectively for the subgroup of LN+ (*N* = 103) and LN− (*N* = 116) patients. For the set of patients (*N* = 59) with available Odx scores, we applied the Trial Assigning Individualized Options for Treatment (Rx) (TAILORx) trial recurrence score categorization^33^ (low: <11, intermediate: 11–25, high: >25) to assign the patients into the low (low recurrence risk), intermediate (ambiguous recurrence risk) and high (high recurrence risk) Odx risk categories. The KM curves were generated for the patients with intermediate Odx category to evaluate whether M_CFOD-TS_ could provide significant prognostic value for the set of patients whose recurrence risk could not be unequivocally interpreted by Odx.

Univariate Cox analysis was performed on clinicopathological variables and Odx risk category to evaluate if any of those clinical routinely examined parameters or the widely used gene test was prognostic of DFS on S_t_ or S_v_. Additionally, we used multivariate Cox regression analysis to evaluate the independent prognostic significance of M_CFOD-TS_ by adjusting the impact of the clinicopathological variables on DFS. Multivariate analysis was performed on the clinicopathological variables which were available for more than two thirds of patients in each cohort (S_t_ or S_v_).

### Enrichment analysis of biological pathways

Histogenomics analysis was performed by investigating the association between biological pathways and CFOD-TS utilizing the normalized Messenger Ribonucleic acid (mRNA) expression data obtained from GDC data portal on TCGA BRCA (S_t_). Histogenomics analysis was, however, not conducted on ECOG 2197 (S_v_) due to lack of corresponding genomic data. Wilcoxon Rank Sum Test (WRST) was first used to identify the genes significantly differentially expressed between R_M_^H^ and R_M_^L^ groups. The identified gene set was in turn taken as the input of Gene Ontology (GO)^51,52^ to identify the associated GO molecular pathways whose member genes were overrepresented in this gene set. After reviewing the list of the identified GO pathways, we selected the ones potentially representative _of_ biological processes underpinning the collagen fiber organization in tumor-associated stroma. The single-sample Gene Set Enrichment Analysis (ssGSEA)^53^ was then used to generate an enrichment score for each of the molecular pathways, assessing the activity level of the pathway in which the member genes were coordinately up- or down-regulated. Unlike GSEA working at the level of sample population, ssGSEA was able to calculate a separate enrichment score for each individual patient. The Pearson correlation was then used to assess the correlation between the enrichment score and the M_CFOD-TS_ derived risk score.

### Reporting summary

Further information on research design is available in the Nature Research Reporting Summary linked to this article.

## Supplementary information

Supplementary Materials

Reporting Summary

## Data Availability

The data generated and analyzed during this study are described in the following data record: 10.6084/m9.figshare.14755374^54^. The majority of the data underlying the claims of this article are openly available in the ten files included in the data record. The remaining data are in the following two files: ‘pred_risk_label_survival_data(Sv).xlsx’, ‘clincopathological_survival_data(Sv).xlsx’. These two files are housed on institutional storage and are not publicly available in order to protect patient privacy as informed consent to share participant-level data was not obtained prior to or during data collection. Requests for access to these data should be directed to the corresponding author.

## Code availability

The code developed and utilized during the current study are available from the corresponding author on reasonable request.
