# Supplementary material for: Collagen fiber orientation disorder from H&E images is prognostic for early stage breast cancer: clinical trial validation
Source: NPJ Breast Cancer. 2021 Aug 6;7:104. doi: 10.1038/s41523-021-00310-z (PMC8346522; doi:10.1038/s41523-021-00310-z)
Supplement: Supplementary file 1 — Supplementary Materials [file 41523_2021_310_MOESM1_ESM.pdf]

Patient inclusion/exclusion flowchart:

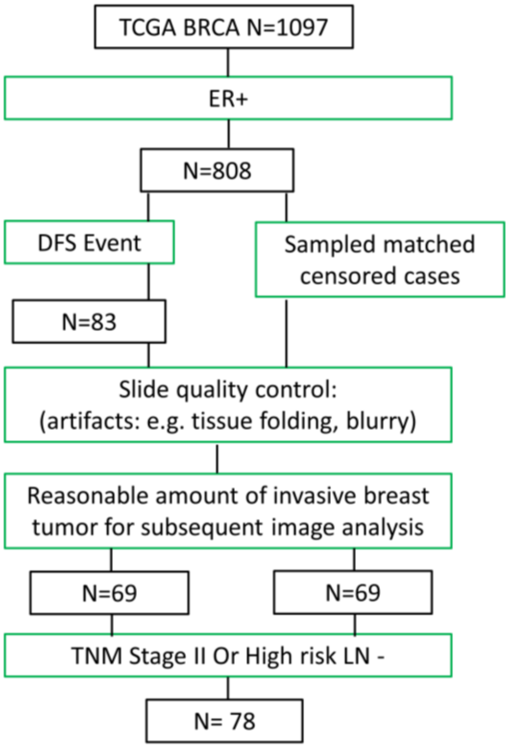

Abbreviation: LN: lymph node; ER+: estrogen receptor positive; TNM: Tumor, Node & Metastasis.

Supplementary Figure 1: Flowchart of patient inclusion/exclusion criteria on TCGA BRCA (S_t) and ECOG 2197 (S_v)

Calculation of Collagen Fiber Orientation Disorder in Tumor-associated Stroma (CFOD-TS):

The linear structure detected in section *Detection of collagen fiber orientations in tumor-associated stroma* in the main manuscript was denoted as vector V_i, which has an associated orientation $\Theta(V_i)$ and length $l(V_i)$. The collagen fiber orientation co-occurrence matrix was generated by calculating the frequency of a vector with orientation *x* co-occurs with a vector with orientation *y* weighted by the corresponding vector length. Supplementary Formula 1 shows how the value in the cell of row x and column y of the co-occurrence matrix was calculated. The matrix was further normalized by dividing the entire matrix by the total summed matrix values.

$\Theta(V_{x1}), \Theta(V_{x2}), ...\Theta(V_{xn}) == x$, $\Theta(V_{y1}), \Theta(V_{y2}), ...\Theta(V_{yn}) == y$.

$$P(x,y) = (l(V_{x1}) + l(V_{x2}) + ...l(V_{xn})) * l(V_{y1}) + l(V_{y2}) + ...l(V_{yn}))$$ Supplementary Formula 1

Following the generation of the orientation co-occurrence matrix, entropy of collagen fiber orientations (CFDO-TS) was calculated by Supplementary Formula 2. N_g is dimension of the matrix (18 in our case) and *i* is discretized angular bin from 0 to 17.

$$-\Sigma_{i=0}^{N_g-1} p_{x-y}(i) log(p_{x-y}(i))$$ Supplementary Formula 2

List of extracted CFOD-TS descriptors

	Field Of View (size of tumor neighborhood where a CFOD-TS descriptor was calculated: um)								
Whole tumor region	50	75	100	125	150	175	200	225	250
Edge of Tumor	50	75	100	125	150	175	200	225	250

Supplementary Table 1: 18 CFOD-TS descriptors were extracted. Descriptors highlighted in red were the features identified by the elastic net regularized Cox regression model.

Experiments with respect to effect of region of interest (entire tumor / tumor edge) and FOV on CFOD-TS

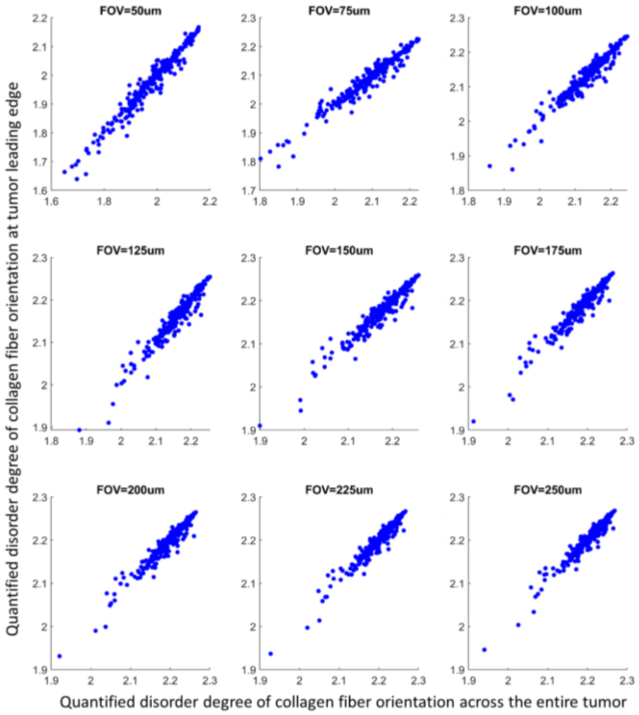

Supplementary Figure 2: Correlation between quantified CFOD-TS in the entire tumor (x axis) and that at the tumor leading edge (y axis) across nine different FOVs (tumor neighborhood size :50 ~ 250 um) on S_t+S_v. Each data point represents the averaged CFOD-TS under the corresponding specified FOV for each individual patient; a higher value indicates a higher degree of collagen fiber orientation disorganization.

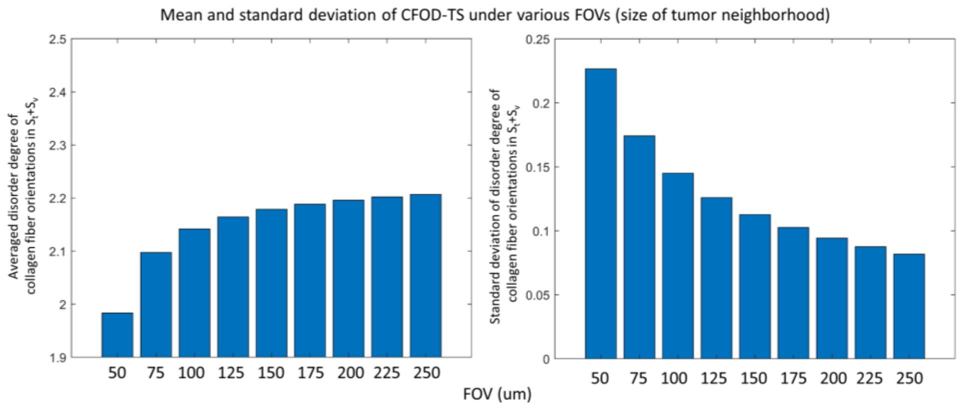

Supplementary Figure 3: The mean (left) and standard deviation (right) of quantitative measurements of CFOD-TS across the entire tumor for nine different FOVs (sizes of tumor neighborhood).

List of relevant molecular pathways

**Pathways related to extracellular organization**
positive regulation of extracellular matrix organization
positive regulation of supramolecular fiber organization
regulation of actin cytoskeleton organization
regulation of actin filament bundle assembly
regulation of actin filament-based process
regulation of stress fiber assembly
**Pathways related to development and division of tumor cells**
cell cycle arrest
cell cycle process
regulation of cell cycle
regulation of cell development

Supplementary Table 2: Molecular pathways relevant to extracellular organization / development and division of tumor cells were identified from the output list of Gene Ontology analysis.

Supplementary List 1: List of identified genes:

CEACAM7	RNF24	ATL2	PNISR	ACKR2
FAM76A	POFUT1	LGALSL	H3F3B	BOD1
GPRC5A	MANBAL	SLC17A5	REEP2	KLHL3
USP2	DHX35	RCL1	PRMT7	TMEM181
FLT4	MCF2	NUP43	ALDH3B2	ZAN
PI4K2B	RS1	TAF12	VSTM2L	STAR
SPATA7	ACP5	ADRA1A	STOML3	GOLGA7
GPM6B	PIEZO1	LRAT	SLC39A11	C9orf3
ARID1B	CTSH	KIAA0087	SLC2A11	HMCN2
HEXB	GABPB1	NT5C3A	UNC79	PARD3
TRAPPC3	CKM	POLM	PRPF38B	NPFFR1
GINM1	AMH	RAMP3	YWHAQ	TCF7L2
PKP2	CLEC4M	TTPAL	FAM189A2	CCDC102B
SNRNP40	AKAP8	KCNK15	HNF1A	ANKRD50
DMRT3	ZNF419	CRISP2	HEY2	GABRA2
EYA2	CYTH2	TRERF1	MICAL1	SACS
MSANTD3	RASIP1	SLC17A1	SP110	SLC30A6
TFE3	TFPT	SOX4	HLX	RAB3C
MAST4	DUS4L	WNT1	WDR38	MED21
CHFR	CBLL1	SOX9	APTX	ADGRA3
MOV10L1	GCK	PSMB2	GRHPR	LURAP1L
SMARCE1	ENG	PROZ	TMEM63B	GRAP
TUBE1	NPM3	IRF3	TTC29	CHST9
ITGA8	RND2	EMG1	SLC28A2	TNIK
TDRD3	MAP2K6	CFP	PPM1B	CCDC173
B4GALT1	PF4V1	PNKD	PDE5A	PPEF2
AAMDC	AREG	LRFN1	CCNG2	MED19
CNOT3	MADD	BAIAP2L2	PAPSS1	ADGRG4
SMOX	VWF	IFT22	FBN2	MUM1L1
FETUB	WNT5B	SMO	AMDHD1	PANK4
NLRP1	SLC22A2	SAFB2	MAP3K12	LRRC43
CAPN3	CUL9	USHBP1	METTL21C	CATIP
PGC	UNC5A	UBAC1	PELI2	TSPAN33
HSD3B7	MORC1	SAG	VPS53	WNT9B
MADCAM1	TFCP2L1	CCDC62	RHBDL3	SYNJ1
PPIL2	OTOF	ZNF331	SYPL2	CES5A
RSPH14	MECR	EDA2R	ANP32E	TGM7
UPK3A	OSCP1	RFTN1	SYT2	CARMIL2
RIMS4	RRAGC	NAPSA	NPHP1	ZBTB8A
BMP7	RCAN3	ACAP3	TMEM177	CLDND2
GID8	PPP1R8	ENOSF1	ZNF385B	PAQR4

PEF1	TTLL6	TSHZ1	MORN2	RF00019
WNT4	TRIAP1	TRNAU1AP	C11orf95	RF00019
IL23R	FAM86JP	CSNK1A1L	LINC00265	RF00019
NTNG1	KRT20	CYP8B1	CLEC2A	RF00264
CCDC138	BCL2L1	BHLHA15	NANOS2	AL626787.1
CFAP221	RAB4B-EGLN2	SSTR2	SERPINA5	BTBD7P1
GABRG1	UTF1	OR56B4	CFAP77	C6orf99
MRPS18C	ZNF57	SAGE1	CYP51A1-AS1	AL354984.1
LMOD3	ANKRD20A4	ANKRD62	TMCO2	GLYATL3
STT3B	SERHL	SLC35C1	NYX	ZDHHC18
C4orf36	COPRS	WRB	ANKDD1B	NOTCH4
DCLK3	BPGM	TDRKH	STK31	C6orf15
MTHFD2L	SMPDL3A	RAD51B	TTC30B	ACOXL-AS1
FBXO40	AGAP5	TMIGD1	IGF2R	LINC01123
ELOVL7	CBWD1	LHFPL1	IFNL3	AL590399.1
CDC20B	COQ2	ANXA2	ZNF841	TMEM88B
TRA2A	FAM46D	C1orf116	HRNR	MT1A
GALNT10	RPL35P9	RPSAP19	FCHSD1	HNRNPA1P20
HCN1	SNAPC5	HMGN3P1	TMEM239	C9orf92
HNF4G	MYO1H	FAM167B	YTHDF2	MUC19
SPIDR	ZNF266	C15orf32	MAFK	BX890604.1
NOS3	ZG16	LINC00518	DDX39B	RP9P
CCDC171	CTBP2	ACTG1	MMP17	C22orf42
ARMC3	PDIK1L	TBX1	PLXNB3	SERPINB4
PHOX2A	MARCKSL1	NCMAP	MIR148A	GOLGA8O
CMTM5	PARL	SLITRK6	MIRLET7G	AC022007.1
USP54	PCCA	PRSS55	RF00019	RF00019
ZSCAN21	HIGD2B	LINC00313	RNU6-1128P	RF00019
SGSM1	C1orf127	IFNLR1	RNU6-1079P	SNORA70F
PRR15L	CHST1	SH3BGR	RNU6-942P	RF00019
ZNF641	LSM1	MORN5	SNORA73B	RNU1-67P
TUBA1A	PRIMA1	EP400P1	RNU6-485P	RF00019
SLC43A2	AC007160.1	SETD4	RNU6-618P	MIR641
AC011511.1	ZNF404	CIDECP	RNU6-137P	MIR558
POLR2G	SCAND2P	ZNF75D	RNU6-824P	MIR642A
PTGDR	SHMT1	GOLGA8R	RNU6-595P	MT-TW
LDLRAD4	YIPF7	MYT1L	RF00418	IGKV1D-42
SLC35G2	SHISA3	UBE2H	RNA5SP245	IGLV5-37
IL13	AC068473.1	HPDL	RNA5SP202	IGLV2-23
NPR1	KLHDC7A	POFUT2	RNU4-40P	IGLV2-14
HINT1	RPS2P28	CDHR4	SNORA55	IGLC2
LINGO1	AL162457.1	SHISA7	VTRNA1-2	IGLC7
WNT10B	LACC1	RINL	RF00019	TRAV23DV6
NSG2	GIPC3	HNRNPA1P61	RN7SKP249	TRAJ34

IGHD3-22	AL356234.1	GAPDHP54	AL590609.2	SETP5
RNU6-930P	YAP1P1	AC000124.1	HERC2P4	AL591212.1
RNA5SP219	ASS1P1	CACTIN-AS1	FANCD2P2	RPL10P12
RNU6-780P	PKMP3	OSTCP8	LINC01048	RNF219-AS1
RNU6-796P	RPL5P18	AL358075.1	PA2G4P4	RHOXF1P1
PFN1P11	MIR1302-8	LBX1-AS1	AC011005.1	AL359845.1
GAPDHP71	RNU4ATAC16P	ST7-AS1	LINC01001	MCIDAS
AC093816.1	MIR1289-1	AL445686.1	AC093423.2	LINC01287
YBX1P6	CCNL2	CDK8P2	SDAD1P2	SLC25A25-AS1
ZBTB9	RNU6-1156P	AC254562.1	AL161636.2	TPT1P9
AL807752.1	RNU6-519P	AL160163.1	PSAT1P3	AC090505.1
RPL7AP4	RNA5SP508	MIR4290HG	RPEP3	AL022329.1
RAB5CP1	CDRT15	AL121877.1	RPL23AP15	LINC01777
AC098614.1	ROR1-AS1	ALG1L8P	AC103563.2	AL360181.2
EEF1B2P2	AL008723.1	ATP5F1AP2	AL078587.1	JARID2-AS1
NPM1P33	PDCL3P6	AL353597.1	GYG1P3	AC073150.1
RPS10P2	AC103563.1	AL357140.2	Z97353.1	AC007283.2
AC019080.1	LINC00853	AC004941.1	ARHGEF9-IT1	LINC01647
LINC02418	BEND3P2	AL022341.1	AL354754.1	CPB2-AS1
AL096701.1	THRB-IT1	AL158070.1	IMPDH1P10	LINC01553
AC002504.1	RPS4XP16	LINC01546	AC239803.2	AL138737.1
RPSAP53	SRP14P1	AL137025.1	AL138902.1	AL603882.1
EFCAB9	PRRT4	AL359091.2	AL390728.5	FMR1-IT1
LINC02085	AC004552.1	AC073583.1	U52111.1	AC005220.1
ZSWIM8	AL450996.1	Z82198.1	AL034417.1	ATG10-IT1
AL391419.1	RPS12P26	ZNF883	AL157400.2	AC099335.1
PPIAP79	AL591178.1	AL513480.1	AC018742.1	AC002401.1
NPIPA7	TGIF2P1	AL356310.1	AC095030.1	INTS6-AS1
ACSM4	CCRL1P1	EXOSC3P1	RAB1C	AL157884.3
AL645728.1	LINC01107	IGHV3-47	AL358075.3	RPL23AP95
OR7E122P	AL158824.1	AL356583.2	CTAGE7P	OR2R1P
AL353795.1	AC073325.1	LINC00710	AL117336.1	RPL23AP23
SCRT2	AC002480.1	AL807752.2	HMGB1P31	AC130710.1
EFCAB8	LINCMD1	CICP3	AC092447.5	AL159174.1
AL356057.1	RPS27P16	LYST-AS1	STXBP5-AS1	FUCA1P1
AL158066.1	AL139246.2	AL450327.1	AC007690.1	EIF3IP1
AL021407.3	AL592310.1	AL354707.2	GAS6-AS1	AL596218.1
HIST1H2APS3	MTND4P32	LINC02526	SLC26A4-AS1	AL035706.1
SPTLC1P2	TRIM64FP	AC110615.1	AL031587.2	AC073254.1
DBIP1	RPS12P5	UBE2Q1-AS1	AL591479.1	ZBTB40-IT1
TUBB8P2	MRPL35P3	SNRPFP2	UFL1-AS1	AC008074.2
NPM1P37	RPS3AP38	CCNB1IP1P1	ZDHHC20P1	SRRM1P3
NPM1P38	IFNA20P	MAS1LP1	AL356859.1	RAI1-AS1
BTBD10P2	AC060234.1	LINC01433	AC092159.3	AC246785.3

AL645634.2	IGHJ3	AC139495.3	AF131215.4	MC1R
AL157714.2	RPL23AP66	AC104619.3	C1DP5	AL049871.1
RPSAP15	RN7SL861P	AC053527.1	AP002807.1	AL049870.3
SHISA9	RN7SL614P	PURPL	AC018523.1	COX5AP2
AL031726.1	AGAP13P	AC095057.2	AC087442.1	AL132642.1
GGTA2P	AL358394.2	AC068658.1	DEFB130C	DOCK11P1
AC073130.1	AC080013.2	AC091435.2	AP000941.1	PTBP1P
AL627309.1	RPLP0P2	REELD1	DDX18P5	ANKRD34C-AS1
AC009948.2	EEF1A1P10	AC015795.1	AP002490.2	IGHV2OR16-5
AC104978.1	RN7SL78P	IGKV2D-40	AP003057.1	AC242376.1
POLR2LP1	TUBA4B	AC020661.1	AC103843.2	AC051619.3
HMGN2P17	RPL13AP2	AC004069.1	AC005833.1	CERNA1
RNA5SP103	IGKV2-28	SHANK3	AC091564.6	AC009065.1
SCARNA7	RPS12P20	ZNF550	KRT8P11	AC106820.3
RNU1-13P	TMEM225B	AC079340.2	AC091814.1	AC138028.4
RN7SL546P	AL021707.5	AC079380.1	AC024901.1	ITFG1-AS1
KCNMB3P1	AC022795.1	AC063919.1	AC135586.1	ACTG1P16
AL442663.1	ASLP1	IGKV1D-39	GCSHP4	AC009088.2
AL513122.2	ARNTL2-AS1	PTX4	AC025423.1	AC120024.1
AL121776.1	LINC00461	RNU7-84P	AC027544.2	LINC00562
AC068760.1	LINC02211	RNVU1-2	AC244131.1	AL360014.1
RN7SL273P	AC006299.1	RNA5SP243	LINC00508	ATP2A1-AS1
OR9A3P	SOCS2-AS1	LINC01845	GPR142	AC011904.1
RPSAP12	LY6E-DT	SERPINE3	AC087241.3	TPRKBP2
RN7SL146P	AC037459.1	TRNP1	PGAM1P5	AC237221.1
AL122013.1	CYP4F27P	AC103957.1	AC126178.1	AC004158.1
RN7SL116P	AC096733.1	AC025871.1	AL139020.1	ANKRD20A1
AC093010.1	AP000344.2	AC021744.1	AC011603.1	VPS35P1
AC092919.1	PCDHGB8P	AC022217.3	AC073525.1	HCCAT5
PCDHGC5	AL356235.1	IGHV3-60	AC025031.1	AC074051.4
AC015908.1	AC021146.2	PKMP4	AL157935.2	LINC02544
RPL7P41	EGFLAM-AS2	IGLC5	OR7E47P	LINC00563
RPL21P122	AC008588.1	AC016885.2	AC009248.1	AC090651.1
IGKV2-24	HNRNPA1P56	LINC02365	AC026111.1	AC022167.4
AC104411.1	AC037441.1	OR10Y1P	AC093014.1	AC005086.1
AC079944.2	ICE2P1	CHKB-CPT1B	NUTF2P2	AC093849.1
AL021937.4	LINC01411	AC022762.1	AL591767.1	AC105137.1
AC093583.1	TERF1P3	TMX2-CTNND1	LINC00641	DNAAF4-CCPG1
AC022400.3	AC112206.2	AP002336.2	AC007686.2	AC009407.1
C3orf67-AS1	AC083829.1	AP003119.1	LINC02295	AL353708.2
AF107885.1	AC004704.1	RTL1	AC011448.1	AC130462.1
MRE11P1	TERB1	AC087379.2	AL357093.2	AL391840.1
AC093627.6	AC011396.2	AC013799.1	AL359792.1	SNORD3B-2
AC107464.1	YJEFN3	AP001267.1	LINC00520	AC010653.1

LINC01978
AC007952.6
AC116914.2
EIF4A1P9
AC015853.2
AC036164.1
AC119396.1
AC090371.2
AC100832.2
RN7SL850P
AC073508.2
AC005899.1
MIR4701
MIR3125
AC006270.1
AC016876.2
AC097641.1
MIR3613
AP001496.2
C1QTNF1-AS1
AC015917.2
AP005263.1
AC005828.4
AC104996.1
AP005329.2
AC100778.3
AP001099.1
MIR4725
SRP68P1
MIR744
AP000919.3
AC011731.1
AC018521.6
RN7SL123P
AC068473.2
AC119403.2
AC006213.2
AP001120.2
AC008770.2
AP001198.1
AC012615.6
AP005264.3
AC105105.1
AC020911.2
AC005702.1
AC138474.1
AC005180.2
AC008735.2
AC027514.2
AC011477.5
HMGB2P1
AC011468.2
EIF1P6
AC010616.1
BNIP3P30
AC022154.1
AC010524.1
BNIP3P22
AC010271.1
AC025588.1
AC003002.2
AC005523.1
CKS1BP3
AC011462.3
PTOV1-AS2
AC011497.2
AC005515.2
AC010326.2
AC010320.4
AL031429.2
AC010530.1
AC079880.2
STX8P1
IGHV1OR15-4
HMGN3-AS1
IGHV3OR16-12
IGHV3OR16-9
URGCP-MRPS24
AC073657.2
AP000526.1
AC090578.1
BOLA2P2
AC010201.1
AL355297.3
IGHV1OR15-3
LINC02104
AL392112.1
BBIP1P1
AL355297.4
AL603832.3
AC098614.4
AL137784.2
AC013472.3
AL139423.1
AL451064.1
AL645940.1
AL353588.1
AC010857.1
MUSTN1
AL592494.3
AP005131.7
AC074044.1
AC084018.1
AP000347.2
C2orf15
Z73429.1
AC016747.3
AC099329.2
AC099676.1
RF00561
AC096708.3
AC005695.3
AC084757.4
AC113385.2
AC022079.1
FAM27E3
AC138649.1
AC084018.2
RN7SL625P
AL157834.3
AC068473.5
AC087588.2
MIR6750
AL031710.2
AC241952.1
AC073578.2
AC009120.5
AC025253.1
SPDYE19P
AC012645.4
FAM27D1
CU633906.1
MIR6793
BX005040.1
HTR1DP1
AC008083.3
AC010999.2
HERC2P2
DACH1
AC004466.3
AC137834.2
MIR8071-2
RF00017
MIR8058
GPR179
MIR6165
AC243571.2
AC007996.1
MIR7845
AC008406.3
AC243773.2
AC139100.2
AC012366.1
AL354811.1
AL031320.2
CHMP1B2P
DHRS11
RF00017
AC145207.9
AC087821.1
CR381670.1
AC079228.1
AC093909.6
AC025678.3
AC005785.2
AC122688.3
AL049776.1
AC135584.1
AP000350.7
AL162426.1
AC002044.2
AL358613.2
AC012313.10
AC020978.9
AL451064.2
AC067931.1
AL050303.4
AC093323.2
AL353597.3
AC117503.3
AC116407.3
AC020661.4
AC092653.2
AC010608.3
AC023632.6
AC026471.6
AC025262.2
AC124303.2
AC061999.1
AC092159.4
AC012186.3
AP000648.3
AL109806.1
FP236383.1
LINC00628
RF00134
AL512506.3
